# Associations of psychological status and ultrasonic characteristics of thyroid nodules in adults during the COVID-19 pandemic

**DOI:** 10.3389/fpsyg.2023.1202122

**Published:** 2023-07-14

**Authors:** Zhengwu Lei, Zhongxiang He, Ying Mei, Xiaoya Qi, Pingping Yu, Guoqiong Xu, Hongfeng Cheng, Ruixue Bai, Jing Deng

**Affiliations:** Health Medical Center, Second Affiliated Hospital of Chongqing Medical University, Chongqing, China

**Keywords:** COVID-19, depression, anxiety, stress, ultrasonic, thyroid nodules

## Abstract

**Background:**

The morbidity of thyroid cancer has been increasing in the last decades all over the world. In addition to the more sensitive thyroid nodule screening technology, several social and environmental factors might represent credible candidates for this increase. They include psychological stress, lifestyle-associated risk factors, nutritional deficiencies, and environmental pollutants. Foremost, psychological stress had gained high interest as a possible promoter and a modifiable risk factor for thyroid nodules in recent years. The present study was to investigate the clinical characteristics and psychological status of the population during the peak of coronavirus disease 2019 (COVID-19) and assessed the association of psychosocial determinants and the ultrasonic characteristics of thyroid nodules.

**Methods:**

In this cross-sectional study, 490 adult subjects who had received at least two doses of COVID-19 vaccine and were not infected with COVID-19, and did not know whether they had thyroid nodules, received thyroid color ultrasound examination and psychological questionnaire survey. Depression, anxiety, and stress were assessed using Depression Anxiety Stress Scales-21 (DASS-21). Sleep quality was rated using the Pittsburgh sleep quality index (PQSI). The characteristics of 243 subjects with thyroid nodules were described and recorded in detail by thyroid color ultrasound, and the correlations between anxiety, depression, sleep quality, clinical indicators, and thyroid nodule ultrasound characteristics were analyzed. Associations between psychological status (mutually adjusted predictors) and ultrasonic characteristics of thyroid nodules (outcome) were modeled using binary logistic regression controlling for sex, age, BMI, TSH, FT3, and FT4.

**Results:**

Depression was positively correlated with thyroid hypoechoic nodule (OR = 3.720, 95%CI 1.615–8.570), microcalcification of thyroid nodule (OR = 3.638, 95%CI 1.476–8.966), the aspect ratio of thyroid nodule>1 (OR = 3.860, 95%CI 1.052–14.161), the unclear boundary of thyroid nodule (OR = 4.254, 95%CI 1.359–13.312), and the irregular edge of thyroid nodule (OR = 4.134, 95%CI 1.810–9.439). Anxiety was positively correlated with microcalcification of thyroid nodules (OR = 4.319, 95%CI 1.487–11.409). Stress was positively correlated with thyroid hypoechoic nodules (OR = 4.319, 95%CI 1.487–11.409), microcalcification of thyroid nodules (OR = 2.724, 95%CI 1.038–7.151), and the irregular edge of thyroid nodules (OR = 2.478, 95%CI 1.077–5.705).

**Conclusion:**

This study demonstrates that depression, anxiety, and stress were associated with the morbidity of thyroid nodules and thyroid ultrasound characteristics. During COVID-19, people’s negative emotions increased significantly compared to before. Negative emotions might be harmful to thyroid health. Therefore, during periods of high stress, strategies to prevent psychological problems should be implemented to improve thyroid health.

## Introduction

With the COVID-19 pandemic outbreak at the end of 2019, the disease rapidly spread worldwide. In the initial period, China was the most impacted by this pandemic, and there was an accompanying epidemic of anxiety and depression nationwide (Wang et al., 2020; Zhou et al., 2020; Wang et al., 2021).

Several studies have explored anxiety, depression, and quality of life during the COVID-19 pandemic in a variety of populations, including the general population (Shi et al., 2020; Zhou et al., 2020; Wang et al., 2021), healthcare workers (Lai et al., 2020; Hummel et al., 2021; Mattila et al., 2021), medical students (Harries et al., 2021; Li et al., 2021), cancer patients (Wang et al., 2020; Kassianos et al., 2021; Kim et al., 2022), and postoperative surgical patients (Doglietto et al., 2020; Chen et al., 2021). The majority of these study populations experienced negative emotional states during the pandemic.

Bad emotions were closely related to the occurrence and development of many diseases, especially the diseases of the endocrine system (Nagaraja et al., 2016). As a common and frequently occurring disease of the endocrine system, thyroid nodules (TN) were the pathological changes of thyroid cells that grow abnormally in local areas. In the adult population, the physical examination could only detect 5 ~ 7% of the morbidity rate, while ultrasound could detect 20% ~ 76% in the same population, which was consistent with the data found in the autopsy (Zamora et al., 2022). At present, the prevalence e of thyroid nodules and thyroid cancer was increasing and becoming younger all over the world (Francis et al., 2015).

Emotions were the reflection of the stress response, which in turn affected the endocrine system. The hypothalamus pituitary thyroid axis had been identified as one of the neuroendocrine systems related to stress response. From the perspective of psychosomatic medicine, persistent negative emotions could lead to physical diseases, including thyroid diseases and tumors. Therefore, a large number of studies had begun to discuss the pathogenesis and prognosis of thyroid diseases from a psychological perspective (Ritchie and Yeap, 2015). The fast-paced life, high work pressure, and lack of sleep made the body often in a chronic stress state. Especially during the epidemic period of COVID-19, psychological burden and alexithymia would cause anxiety and depression in the population, which might further lead to the disorder neuroendocrine-immune system and thyroid hormone regulation, and could eventually cause thyroid disease and nodular goiter.

Among the common endocrine pathological system diseases, thyroid dysfunction was closely related to anxiety, depression, sleep disorders, etc. For example, the prevalence of psychological negative emotions in patients with hyperthyroidism (Hu et al., 2013) and Hashimoto’s thyroiditis was high (Giynas Ayhan et al., 2014). Research showed negative emotions could impair immune function (Sephton et al., 2009). Graves’ disease and Hashimoto’s thyroiditis were both autoimmune diseases. Due to the immune function disorder caused by negative emotions, the thyroid auto thyrotropin receptor antibody, antithyroid peroxidase antibody, and antithyroglobulin antibody increase, which continuously attacked thyroid cells, leading to chronic inflammation. Chronic inflammation could not only lead to thyroid dysfunction, induce hyperthyroidism and hypothyroidism, but also lead to abnormal proliferation, division, and replication of thyroid cells, forming thyroid nodules, and even thyroid cancer (Ferrari et al., 2020).

Some studies believed that common negative emotions such as anxiety. Depression and psychological stress were one of the risk factors for thyroid nodules (Fang et al., 2014), and some researchers had studied negative emotions as independent risk factors for thyroid cancer. They believed that negative emotions, such as psychological stress, would cause changes in nerve transmission in the cerebral cortex and hypothalamus (Azizi and Malchoff, 2011), directly or indirectly weakening the immune system, leading to an increase in the morbidity of malignant nodules. Few studies had assessed the association between psychological status and the morphology, biochemistry, pathology, and other characteristics of thyroid nodules. Therefore, this study aimed to quantify the association between psychological status and characteristics of thyroid ultrasound characteristics in the population of southwest China during the COVID-19 pandemic, to promote screening and prevention of thyroid nodules, and to further improve thyroid health management.

## Materials and methods

### Study design and participants

We conducted a cross-sectional study and recruited 490 participants from the health management center of the general tertiary hospital located in Southwest China between 1 January 2020 and 31 December 2022. The inclusion criteria were: (Wang et al., 2021) over the age of 18; (Zhou et al., 2020) underwent thyroid color Doppler ultrasonography, blood biochemical index examination, life habit survey, and psychological evaluation; (Wang et al., 2020) the subjects had no history of mental illness, had the ability of independent behavior and volunteered to participate in the study. (Shi et al., 2020) got at least two doses of COVID-19 vaccine. The exclusion criteria were: Wang et al. (2021) known thyroid disease, such as pre-diagnosed nodular thyroid disease, thyroid dysfunction, or under treatment with thyroid hormone or anti-thyroid drugs followed up by endocrinologists; Zhou et al. (2020) receiving psychotherapy or antidepressant and anxiety treatment; Wang et al. (2020) a history of previous neck radiation therapy. Shi et al. (2020) COVID-19 infection.

### Data collection

A standard questionnaire was used to collect socio-demographic characteristics, individual disease history, first-degree family disease history, and relevant lifestyle factors. The DASS-21 questionnaire is a quantitative 21-item screening tool to assess symptoms of depression, anxiety, and stress. The PSQI questionnaire was used to assess the sleep status of participants over the past month. For all individuals, their weight, height, and blood pressure were measured according to a standard protocol and recorded. All anthropometric and biochemical data were collected at the same time. The body mass index (BMI) was calculated from weight and length (kg/m2).

### Laboratory assays

Serum samples were collected by a venous puncture after an overnight fast between 7:00 AM and 10:00 AM. The blood samples were cooled down to 4°C and within 2–4 h they were transported under cooling to a central laboratory that was certified by the College of American Pathologists (CAP). The following laboratory assays were performed: fasting plasma glucose (FPG), total cholesterol (TC), triglycerides (TG), low-density lipoprotein (LDL), and high-density lipoprotein (HDL) were assessed by Hitachi, LABOSPECT 008AS (Tokyo, JAPAN). Thyroid peroxidase antibodies (TPO-Ab) and thyroglobulin antibodies (Tg-Ab) were detected by chemiluminescence (SNIBE, Biolumi 8,000, Shenzhen, China), Thyroid-stimulating hormone (TSH), free triiodothyronine (FT3), and free thyroxine (FT4) levels were measured by chemiluminescent immunoassays (Siemens, Centaur XPT, Erlangen, Germany).

### Thyroid ultrasonography

Ultrasound investigations of the thyroid were performed by two registered physicians who both had a professional certificate for ultrasonography (awarded by the Ministry of Health of China). Ultrasound was performed using B-mode US imaging (MX7, Mindray Shenzhen, P.R. China) with a 13 MHz linear array probe. Observe the echo of thyroid parenchyma (TH), internal echo, number, calcification, aspect ratio, boundary, and shape of thyroid nodules, and record the detection results. The echo characteristics of thyroid nodules were divided into anechoic nodules, mixed echo nodules, hypoechoic nodules, isoechoic nodules, hyperechoic nodules, and multiple echo mixed nodules. The ultrasonic image with space-occupying effect echo was determined as thyroid nodules.

### Definition of variables

This DASS21 comprised three subscales. The anxiety subscale (DASS-21 A): with the following categories for the total score—normal (0–7), anxiety (>7); The depression subscale (DASS-21 D): with the following categories for the total score—normal (0–9), depression (>9); The stress subscale (DASS-21S)—with the following categories for the total score: normal (0–14), stress (>14).

The 19 items of PQSI were divided into seven component scores that reflected the severity of various sleep problems in the following aspects: subjective sleep quality, sleep latency, sleep duration, habitual sleep efficiency, sleep disturbance, use of sleep medication, and daytime dysfunction. The global score of PSQI ranged from 0 to 2 L. The higher the score, the worse the sleep quality.

The following reference ranges for normal thyroid functions were used: 3.1–6.8 pmol/L for FT3, 9.5–24.5 pmol/L for FT4, and 0.35–5μIU/mL for TSH. A value of TPO-Ab >34 U/mL or of Tg-Ab >115 IU/mL was defined as positive. Smoking status was divided into smokers (at least 100 cigarettes were smoked in the past and are currently smoking) and non-smokers, Excessive drinking was defined as alcohol intake ≥210 g per week for men and ≥ 140 g per week for women. Excessive iodized salt was defined as>6 g per day, and Excessive intake of cooking oil was defined as>30 g per day.

### Statistical analysis

**Figure 1:**
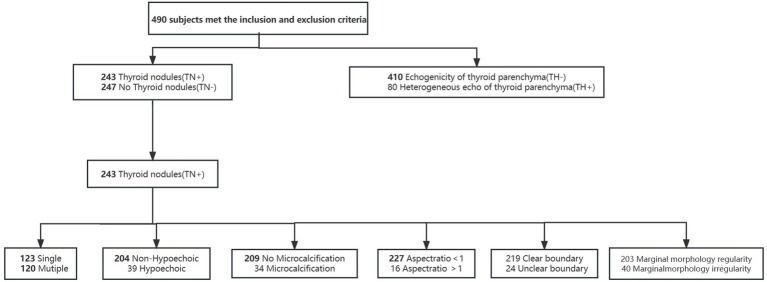
Flowchart for data processing.

Statistical analyses were performed using IBM SPSS Statistics V.23 (IBM Corp, Armonk, NY, USA). Two-tailed *p* values below 0.05 were considered statistically significant. Continuous variables with normal distribution were expressed as the mean ± SD, while non-normally distributed variables were expressed as a median and interquartile range for the 25th-75th percentile. Categorical variables were presented as counts and percentages and used chi-square test for categorical variables. Continuous variables were compared with Student’s t-test. The Mann–Whitney U test was applied to non-normally distributed data. Factors with univariate *p* ≤ 0.05 were included in the multivariable logistic regression analysis. The associations of ultrasonic characteristics of thyroid nodules and psychology status were assessed by binary logistic regression (Figure 1). The ROC curve analysis was used to compare the predictive ability of psychological and clinical characteristics on ultrasonic characteristics of thyroid nodules.

This study included a total of 490 participants, of which 243 had thyroid nodules and 247 did not. On the other hand, the thyroid parenchyma of 80 subjects had heterogeneous echoes, while the thyroid parenchyma of 410 subjects had homogeneous echoes. Compare TN (+) and TN (−), TH (−) and TH (+), and statistically analyze differences in clinical features and psychological states. Further analysis of the ultrasound characteristics of thyroid nodules revealed the correlation between psychological status and thyroid nodules.

## Results

### Baseline characteristics of participants with or without thyroid nodules and with or without the homogeneous echo of thyroid parenchyma

The general demographic and clinical characteristics of all 490 subjects (204 men and 286 women) included in the study were summarized in Table 1. In comparison to those with and without thyroid nodules: 14.8% of the total participants were smokers. 7.8% of those showed excessive alcohol consumption. 64.3% had an average monthly income of less than 6,000 yuan. 12.4% of the study subjects had less than 9 years of education, and 9.6% had taken excessive iodized salt. 14.9% of subjects consumed excessive cooking oil. 34.2% suffered from chronic diseases. Thyroid nodules (TNs) were found in 49.6% and lower in men (39.1%) than in women (60.9%). The proportion of high-risk alcohol consumers was significantly higher in subjects without TNs than in those with TNs (*p* < 0.05). Three metabolic factors were associated with the presence of TNs: a higher level of TG, Glu, and lower TBIL (all *p* < 0.05). Of the five investigated parameters related to thyroid function indexes and autoantibodies, none resulted in statistically significant differences between TN-positive and TN-negative individuals. Among the psychological factors investigated, there were significant differences in the prevalence of depression, anxiety, and stress between the two groups. In comparison to those subjects with and without the homogeneous echo of thyroid parenchyma: a heterogeneous echo of thyroid parenchyma was found in 16.3% and lower in men (18.8%) than in women (81.2%). The proportion of smokers and high-risk alcohol among subjects with the homogeneous echo of thyroid parenchyma were significantly higher than that among subjects with the heterogeneous echo of thyroid parenchyma (*p* < 0.05). Four metabolic factors were associated with the presence of heterogeneous echo in the thyroid parenchyma: lower levels of UA, CR, Hemoglobin, and higher levels of TSH (all *p* < 0.05). It seemed that the proportion of chronic diseases (such as hypertension, diabetes, coronary disease, and chronic hepatitis) in subjects with the heterogeneous echo of thyroid parenchyma was slightly higher than in those with the homogeneous echo of thyroid parenchyma (*p* = 0.051), but unfortunately, there was no significant difference. In the psychological factors investigated, there were significant differences in the prevalence of depression and stress between the two groups. However, there was no significant statistical difference in sleep quality.

**Table 1:** Baseline characteristics of participants with or without thyroid nodules (TNs) and with or without homogeneous echo of thyroid parenchyma (TH).

Parameter	TN(−)	TN(+)	*p*	TH(−)	TH(+)	*p*
*N*(%)	247	243		410	80	
Age(years)	39.06 ± 9.20	42.95 ± 10.46	**0.000**	40.68 ± 10.06	42.55 ± 9.75	0.128
Men(%)	109(44.1%)	95(39.1%)	0.258	189(46.1%)	15(18.8%)	**0.000**
BMI(kg/m^2^)	22.80 ± 3.15	23.27 ± 3.25	0.101	23.13 ± 3.21	22.54 ± 3.17	0.131
Edu years			0.237			0.531
≤9 years	26(10.5%)	35(14.4%)		48(11.7%)	13(16.3%)	
9–16 years	184(74.5%)	181(74.5%)		308(75.1%)	57(71.3%)	
>16 years	37(15.0%)	27(11.1%)		54(13.2%)	10(12.5%)	
SBP (mm hg)	114.5 ± 13.7	116.8 ± 16.0	0.084	115.96 ± 15.02	114.04 ± 14.60	0.293
DBP (mm hg)	69.53 ± 9.95	70.62 ± 11.09	0.254	70.24 ± 10.63	69.20 ± 10.06	0.418
WHR	0.82 ± 0.07	0.82 ± 0.08	0.928	0.82 ± 0.08	0.81 ± 0.07	0.113
Income class (CNY)			0.819			0.362
≤6,000	160(64.8%)	155(63.8%)		260(63.4%)	55(68.8%)	
>6,000	87(35.2%)	88(36.2%)		150(36.6%)	25(31.3%)	
Smoking status(%)			0.955			**0.000**
smoking	35(14.2%)	34(14.0%)		68(16.6%)	1(1.3%)	
Never smoking	212(85.8%)	209(86%)		342(83.4%)	79(98.8%)	
Drinking status(%)			**0.008**			**0.012**
No drinking	220(89.1%)	232(95.5%)		373(91%)	79(98.8%)	
Excessive Drink	27(10.9%)	11(4.5%)		37(9%)	1(1.3%)	
Chronic diseases (%)	81(32.8%)	87(35.8)	0.483	133(32.4%)	35(43.8%)	0.051
Iodized salt intake(%)			0.107			0.644
Low	42(17%)	35(14.4%)		66(16.1%)	11(13.8%)	
Moderate	188(76.1%)	178(73.3%)		303(73.9%)	63(78.8%)	
High	17(6.9%)	30(12.3%)		41(10%)	6(7.5%)	
Oil intake(%)			0.455			0.815
Low	39(15.8%)	39(16.0%)		67(16.3%)	11(13.8%)	
Moderate	176(71.3%)	163(67.1%)		283(69%)	56(70%)	
High	32(13.0%)	41(16.9%)		60(14.6%)	13(16.3%)	
TSH(uIU/ml)	2.63 ± 1.81	2.43 ± 1.33	0.148	2.41 ± 1.23	3.16 ± 2.70	**0.020**
FT3(pmol/ml)	4.46 ± 0.57	4.52 ± 0.50	0.166	4.49 ± 0.53	4.48 ± 0.56	0.936
FT4(pmol/ml)	16.70 ± 1.96	16.77 ± 1.92	0.670	16.74 ± 1.87	16.71 ± 2.30	0.905
TPOAb-positive(%)	40(16.2%)	40(16.5%)	0.936	0(0.0%)	80(100%)	1.000
TgAb-positive(%)	40(16.2%)	40(16.5%)	0.936	0(0.0%)	80(100%)	1.000
γGGT	20(15, 33)	20(15, 35)	0.726	20(15, 34)	18(14, 34)	0.132
ALT	16(11, 26)	17(12, 25)	0.581	17(12, 25)	16.5(12, 22.75)	0.513
AST	19(17, 23)	19(16, 23)	0.415	19(17, 23)	19(16, 22.75)	0.697
TBIL	9.9(7.5, 12.8)	9(7.1, 11.3)	**0.018**	9.65(7.3, 12.225)	8.65(7, 10.78)	0.056
Nucleotidase	3.9(3, 5)	3.8(3.1, 5.3)	0.672	3.85(3.1, 5.2)	3.8(2.93, 5.0)	0.532
UA	362.03 ± 90.7	348.36 ± 92.8	0.100	360.73 ± 92.52	327.15 ± 83.68	**0.003**
Albumin	45.89 ± 2.75	45.46 ± 2.94	0.095	45.75 ± 2.91	45.29 ± 2.56	0.190
Globulin	29.22 ± 4.25	29.12 ± 3.84	0.784	29.04 ± 3.83	29.83 ± 4.99	0.108
Urea	5.41 ± 1.33	5.42 ± 1.34	0.904	5.44 ± 1.33	5.25 ± 1.37	0.238
CR	66.02 ± 17.41	65.08 ± 16.51	0.543	67.05 ± 17.08	57.88 ± 14.12	**0.000**
TG	1.15(0.9, 1.62)	1.29(0.88, 2.15)	**0.048**	1.26(0.9, 1.92)	1.13(0.87, 1.71)	0.181
TC	5.21 ± 1.03	5.34 ± 1.01	0.133	5.27 ± 1.02	5.29 ± 1.06	0.889
HDL-C	1.42 ± 0.36	1.39 ± 0.35	0.335	1.39 ± 0.36	1.47 ± 0.33	0.078
LDL-C	2.95 ± 0.79	3.04 ± 0.82	0.208	3.00 ± 0.80	2.97 ± 0.82	0.778
FPG	4.92(4.62, 5.26)	5.02(4.72, 5.38)	**0.023**	4.97(4.66, 5.32)	4.93(4.7, 5.29)	0.923
Hemoglobin	135.13 ± 13.10	133.46 ± 12.48	0.148	134.85 ± 13.20	131.51 ± 10.22	**0.033**
Platelet	216.46 ± 53.46	220.97 ± 45.10	0.313	219.01 ± 49.06	217.06 ± 51.63	0.747
White blood cell	6.00 ± 1.25	6.00 ± 1.34	0.976	6.03 ± 1.28	5.86 ± 1.39	0.303
Mental health						
Sleep score	6.29 ± 3.35	6.28 ± 3.33	0.991	6.29 ± 3.31	6.26 ± 3.50	0.946
Depression status(%)			**0.000**			**0.000**
No depression	172(69.6%)	114(46.9%)		258(62.9%)	28(35%)	
Depression	75(30.4%)	129(53.1%)		152(37.1%)	52(65%)	
Anxiety status(%)			**0.000**			0.097
No anxiety	175(70.9%)	99(40.7%)		236(57.6%)	38(47.5%)	
Anxiety	72(29.1%)	144(59.3%)		174(42.4%)	42(52.5%)	
Stress status(%)			**0.000**			**0.000**
No stress	197(79.8%)	88(36.2%)		258(62.9%)	27(33.8%)	
Stress	50(20.2%)	155(63.8%)		152(37.1%)	53(66.3%)	

TN(−), no thyroid nodules; TN(+), with thyroid nodules; TH(−), echogenicity of thyroid parenchyma; TH(+), heterogeneous echo of thyroid parenchyma; BMI, body mass index; WHR, waist to hip ratio; SBP, systolic blood pressure; DBP, diastolic blood pressure; TSH, thyroid stimulating hormone; FT3, free triiodothyronine; FT4, free thyroxine; TPOAb, thyroid peroxidase antibody; TgAb, thyroglobulin antibody; FPG, fasting plasma glucose; γGGT, γ-glutamyl transpeptidase; ALT, alanine aminotransferase; AST, Aspartate transaminase; TBIL, total bilirubin; UA, uric acid; CR, creatinine; TG, triglycerides; TC, total cholesterol; LDL-C, low density lipoprotein cholesterol; HDL-C, high-density lipoprotein cholesterol. Bold values indicate *p* < 0.05.

### Clinical characteristics of ultrasound findings in thyroid nodule participants

Tables 2, 3 summarized the clinical characteristics of all 243 subjects diagnosed with thyroid nodules in the study under different ultrasonic manifestations. Albumin levels in subjects with single nodules were higher than those with multiple nodules (*p* < 0.05). The positive rates of FT3, Tbil, anti-thyroid autoantibodies antibody, and Thyroid peroxidase antibody in subjects with hypoechoic nodules were significantly higher than those in non-hypoechoic nodules. In the psychological factors investigated, the depression rate of subjects with hypoechoic nodules was significantly higher than that of subjects without hypoechoic nodules. But there was no significant difference in sleep quality between the two groups.

**Table 2:** Clinical characteristics of nodule number and nodule echo in thyroid nodule participants.

Parameter	Number of nodules		*p*	Nodular echogenicity		*p*
	Single	Multiple		Non-Hypoechoic	Hypoechoic	
*N*(%)	123	120		204	39	
Age(years)	42.41 ± 9.77	43.51 ± 11.13	0.413	42.87 ± 10.57	43.38 ± 9.97	0.778
Men(%)	52(42%)	43(36%)	0.303	78(38.2%)	17(43.6%)	0.288
BMI	23.18 ± 3.05	23.37 ± 3.46	0.656	23.11 ± 3.23	24.12 ± 3.29	0.077
TSH	2.49 ± 1.30	2.36 ± 1.35	0.436	2.43 ± 1.35	2.39 ± 1.22	0.871
FT3	4.55 ± 0.52	4.49 ± 0.47	0.313	4.54 ± 0.50	4.44 ± 0.46	0.269
FT4	16.81 ± 1.71	16.74 ± 2.13	0.755	16.61 ± 1.79	17.63 ± 2.37	**0.002**
TPOAb-positive	22(17.9%)	18(15%)	0.544	29(14.2%)	11(28.2%)	**0.031**
TGAb-positive	22(17.9%)	18(15%)	0.544	29(14.2%)	11(28.2%)	**0.031**
Albumin	45.88 ± 3.06	45.02 ± 2.77	**0.022**	45.36 ± 2.88	45.97 ± 3.23	0.237
Globulin	29.13 ± 3.92	29.11 ± 3.77	0.97	29.23 ± 3.84	28.54 ± 3.82	0.902
TBIL	9.2(7.2, 11.3)	8.85(6.93, 11.45)	0.504	9.39 ± 3.98	11.53 ± 5.76	**0.005**
Mental health						
sleep quality	6.42 ± 3.67	6.14 ± 2.97	0.512	6.22 ± 3.37	6.64 ± 3.14	0.467
Depression status(%)			0.341			**0.001**
No depression	54(43.9%)	60(50%)		105(51.5%)	9(23.1%)	
Depression	69(56.1%)	60(50%)		99(48.5%)	30(76.9%)	
Anxiety status(%)			0.622			0.167
No anxiety	52(42.3%)	47(39.2%)		87(42.6%)	12(30.8%)	
Anxiety	71(57.7%)	73(60.8%)		117(57.4%)	27(69.2%)	
Stress status(%)			0.680			0.062
No stress	43(35%)	45(37.5%)		79(38.7%)	9(23.1%)	
Stress	80(65%)	75(62.5%)		125(61.3%)	30(76.9%)	

BMI, body mass index; TSH, thyroid stimulating hormone; FT3, free triiodothyronine; FT4, free thyroxine; TPOAb, thyroid peroxidase antibody; TgAb, thyroglobulin antibody; TBIL, total bilirubin. Bold values indicate *p* < 0.05.

**Table 3:** Clinical characteristics of nodule calcification, nodule aspect ratio, nodule border, and nodule edge morphology of thyroid nodule participants.

Parameter	Microcalcification		*p*	Aspect ratio		*p*	Boundary		*p*	Marginal morphology		*p*
	(−)	(+)		<1	>1		(−)	(+)		(−)	(+)	
*N*(%)	209	34		227	16		219	24		203	40	
TSH	2.47 ± 1.36	2.17 ± 1.10	0.226	2.45 ± 1.33	2.13 ± 1.29	0.351	2.46 ± 1.33	2.12 ± 1.25	0.241	2.46 ± 1.34	2.24 ± 1.22	0.343
FT3	4.52 ± 0.50	4.51 ± 0.51	0.929	4.53 ± 0.50	4.46 ± 0.48	0.623	4.53 ± 0.49	4.48 ± 0.55	0.691	4.53 ± 0.50	4.48 ± 0.46	0.539
FT4	16.69 ± 1.79	17.31 ± 2.57	0.081	16.67 ± 1.78	18.28 ± 3.03	**0.001**	16.63 ± 1.78	18.13 ± 2.58	**0.000**	16.65 ± 1.84	17.40 ± 2.23	**0.025**
TPOAb(+)	35(16.7%)	5(14.7%)	0.766	35(15.4%)	5(31.3%)	0.099	31(14.2%)	9(37.5%)	**0.003**	29(14.3%)	11(27.5%)	**0.039**
TGAb(+)	35(16.7%)	5(14.7%)	0.766	35(15.4%)	5(31.3%)	0.099	31(14.2%)	9(37.5%)	**0.003**	29(14.3%)	11(27.5%)	**0.039**
γGGT	20(14, 35)	20.5(15, 38)	0.824	20(15, 36)	17(14, 30.5)	0.509	21(15, 39)	15(13.25,23.75)	**0.013**	21(15, 36)	17(14, 29.25)	0.128
ALT	17(12, 25)	18(13, 24.25)	0.801	18(12, 25)	14(10.5, 18.75)	0.164	18(12, 26)	13.5(10.25,19)	**0.026**	18(12, 25)	15(11.25, 21.75)	0.248
AST	19(16, 23)	20(17.75, 22.75)	0.399	19(16, 23)	17.5(15.25, 19.75)	0.069	19(17, 24)	18(15.25,20.75)	0.065	19(16, 23)	19(16, 21)	0.512
TBIL	9(7, 11.1)	9.8(7.58, 12.83)	0.153	9(7.1, 11.4)	9.1(6.83,10.9)	0.915	8.9(7, 11.1)	9.6(7.88,12.23)	0.208	8.7(6.9, 11.3)	10.3(8.63, 12)	**0.044**
Nucleotidase	3.7(3.1, 5.1)	4.8(3.3, 6.13)	**0.017**	3.8(3.1, 5.2)	4.35(2.98, 6.03)	0.393	3.9(3.2, 5.3)	3.4(2.63,3.88)	0.087	3.8(3.1, 5.2)	3.8(3.13, 6.18)	0.350
FPG	5(4.71, 5.31)	5.24(4.87, 6.42)	**0.005**	5.02(4.71, 5.36)	5.085(4.87, 6.38)	0.130	5.03(4.72, 5.38)	4.92(4.65,5.383)	0.467	5.04(4.71, 5.36)	4.915(4.73, 5.42)	0.731
Mental health												
sleep quality	6.29 ± 3.41	6.24 ± 2.90	0.927	6.26 ± 3.38	6.56 ± 2.66	0.730	6.33 ± 3.32	5.83 ± 3.52	0.487	6.25 ± 3.36	6.48 ± 3.23	0.693
Depression status(%)			**0.010**			**0.021**			**0.002**			**0.001**
No depression	105(50.2%)	9(26.5%)		111(48.9%)	3(18.8%)		110(50.2%)	4(16.7%)		105(51.7%)	9(22.5%)	
Depression	104(49.8%)	25(73.5%)		116(51.1%)	13(81.3%)		109(49.8%)	20(83.3%)		98(48.3%)	31(77.5%)	
Anxiety status(%)			**0.001**			0.071			0.224			0.062
No anxiety	94(45%)	5(14.7%)		96(42.3%)	3(18.8%)		92(42%)	7(29.2)		88(43.3%)	11(27.5%)	
Anxiety	115(55%)	29(85.3%)		131(57.7%)	13(81.3%)		127(58%)	17(70.8%)		115(56.7%)	29(72.5%)	
Stress status(%)			**0.041**			0.180			0.099			**0.048**
No stress	81(38.8%)	7(20.6%)		85(37.4%)	3(18.8%)		83(37.9%)	5(20.8%)		79(38.9%)	9(22.5%)	
Stress	128(61.2%)	27(79.4%)		142(62.6%)	13(81.3%)		136(62.1%)	19(79.2%)		124(61.1%)	31(77.5%)	

Microcalcification(−), no microcalcification; Microcalcification(+), with microcalcification; Boundary(−), clear boundary; Boundary(+), unclear boundary; Marginal morphology(−), regularity; Marginal morphology(+): irregularity; TSH, thyroid stimulating hormone; FT3, free triiodothyronine; FT4, free thyroxine; TPOAb, thyroid peroxidase antibody; TgAb, thyroglobulin antibody; FPG, fasting plasma glucose; γGGT, γ-glutamyl transpeptidase; ALT, alanine aminotransferase; AST, Aspartate transaminase; TBIL, total bilirubin. Bold values indicate *p* < 0.05.

The FT4 levels were higher in subjects with nodule aspect ratio > 1, unclear nodule boundary, and irregular nodule edge. The proportion of TPOAB-positive and TGAB-positive in subjects with unclear thyroid nodule boundaries was significantly higher than that in subjects with clear thyroid nodule boundaries (*p* < 0.05). For subjects with unclear thyroid nodule boundaries, the levels of γ GGT and ALT were significantly lower than those with clear thyroid nodule boundaries (*p* < 0.05). TBIL in subjects with irregular thyroid nodule margins was significantly higher than that in subjects with regular thyroid nodule margins (*p* < 0.05). The levels of Nucleotidase and Glu in subjects with microcalcification of thyroid nodules were significantly higher than those in subjects without microcalcification of thyroid nodules (*p* < 0.05). Among the psychological factors investigated, the depression rate was higher if the aspect ratio of thyroid nodules was greater than 1, the boundary was unclear, and the edge was irregular. Subjects with microcalcification of thyroid nodules also had higher rates of depression and subjects with microcalcification and irregular edge of thyroid nodules showed higher stress through scoring.

### The associations between the clinical characteristics and psychological factors of subjects with thyroid nodules

**Figure 2:**
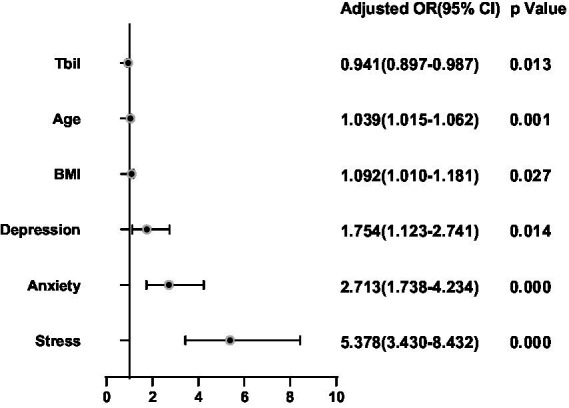
Binary logistic regression analysis for predictors of thyroid nodules. Adjusting for Sex, Age, BMI, TSH, FT3, FT4, Drinking and Smoking.

**Figure 3:**
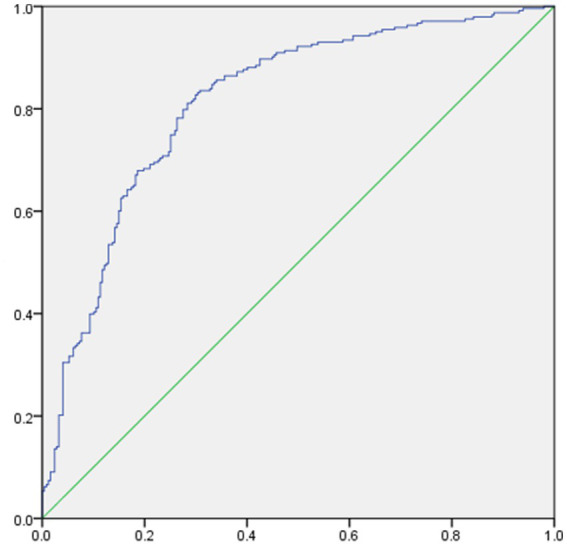
ROC model for predicting factors of thyroid nodules.

It was assessed whether associations existed between the clinical characteristics, psychological factors, and the presence of TNs, for which logistic regression analyses were performed by adjusting for Sex, Age, BMI, TSH, FT3, FT4, Drinking, and Smoking. Age, BMI, Depression, Anxiety, and Stress were positively correlated with the presence of TNs (all *p* < 0.05; Figure 2). Tbil was negatively correlated with the presence of TNs (both *p* < 0.05; Figure 2). The area under the ROC curve showed about 0.813 (*p* < 0.05; Figure 3).

**Figure 4:**
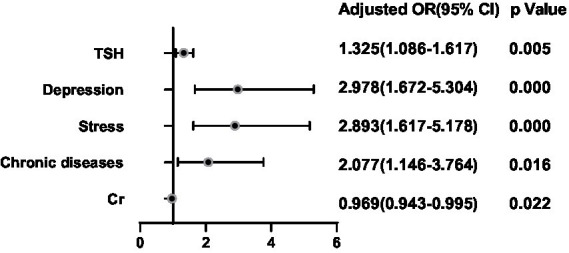
Binary logistic regression analysis for predictors of echo of thyroid parenchyma. Adjusting for Sex, Age, BMI, TSH, FT3, FT4, Drinking and Smoking.

**Figure 5:**
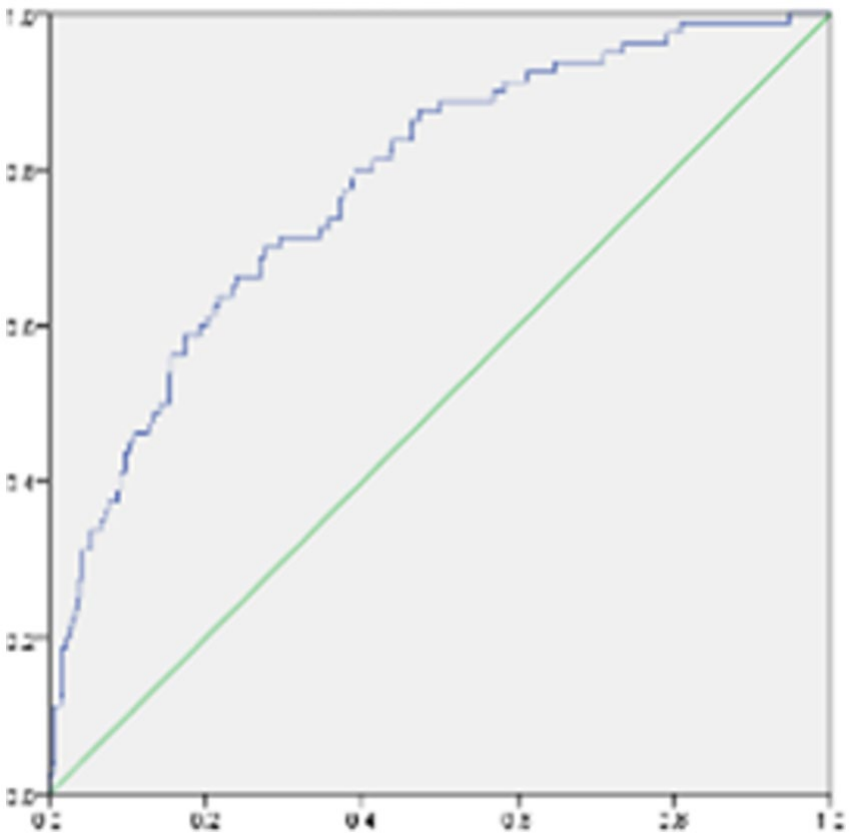
ROC model for predicting factors of echo of thyroid parenchyma.

It was assessed whether associations existed between the clinical characteristics, psychological factors, and the presence of echo of thyroid parenchyma, for which logistic regression analyses were performed by adjusting for Sex, Age, BMI, TSH, FT3, FT4, Drinking, and Smoking. TSH, Depression, and Stress were positively correlated with the presence of thyroid parenchymal echo heterogeneity (all *p* < 0.05; Figure 4). Cr level was negatively correlated with the presence of thyroid parenchymal echo heterogeneity (*p* < 0.05; Figure 4). The area under the ROC curve showed about 0.780 (*p* < 0.05; Figure 5).

### The associations between the clinical characteristics or psychological factors of subjects and the ultrasonic manifestations of thyroid nodules

**Figure 6:**
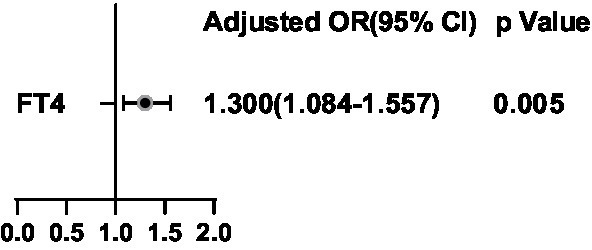
Binary logistic regression analysis for predictors of number of thyroid nodules.

**Figure 7:**
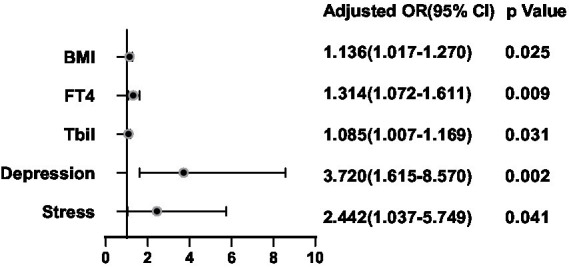
Multivariate logistic regression analysis for predictors of Echo of thyroid nodules.

**Figure 8:**
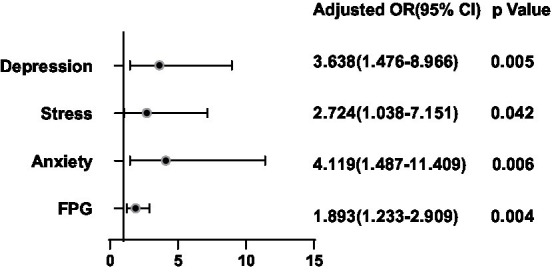
Binary logistic regression analysis for predictors of microcalcification of thyroid nodules.

**Figure 9:**
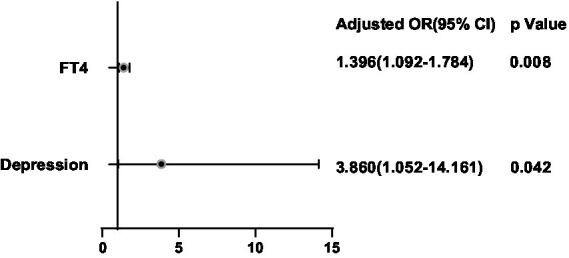
Binary logistic regression analysis for predictors of aspect ratio of thyroid nodules.

**Figure 10:**
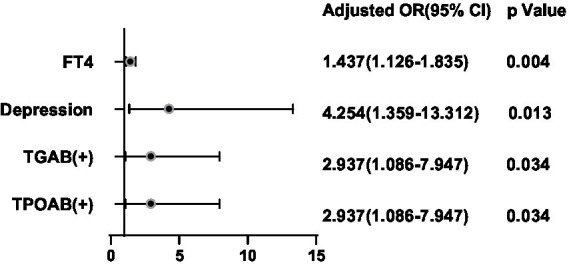
Binary logistic regression analysis for predictors of boundary of thyroid nodules.

**Figure 11:**
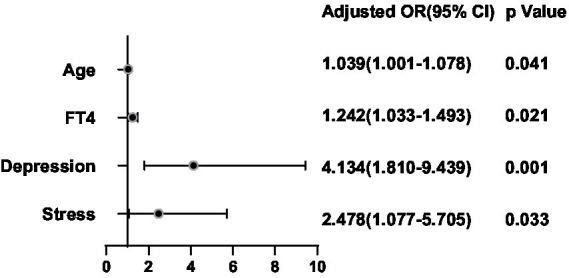
Binary logistic regression analysis for predictors of Marginal morphology of thyroid nodules. Adjusting for Sex, Age, TSH, FT3, FT4, BMI, Drinking and Smoking.

After adjusting for potential confounding factors, a binary logistic regression analysis was conducted between the significant factors in the single-factor analysis and the ultrasonic manifestations of thyroid nodules in the thyroid nodule populations. FT4 was positively correlated with the presence of multiple nodules (OR = 1.300, 95%CI 1.084–1.557, *p* = 0.005) (Figure 6). BMI (OR = 1.136, 95%CI 0.741–0.987, *p* = 0.025), FT4 (OR = 1.314, 95%CI 1.072–1.611, *p* = 0.009), Tbil (OR = 1.085, 95%CI 1.007–1.169, *p* = 0.031), Depression (OR = 3.720, 95%CI 1.615–8.570, *p* = 0.002) and Stress (OR = 2.442, 95%CI 1.037–5.749, *p* = 0.0041) were positively correlated with hypoechoic nodules (Figure 7). Depression (OR = 3.638, 95%CI 1.476–8.966, p = 0.005), Anxiety (OR = 4.119, 95%CI 1.487–11.409, *p* = 0.006), Stress (OR = 2.724, 95%CI 1.038–7.151, *p* = 0.042), and Glu (OR = 1.893, 95%CI 1.233–2.909, *p* = 0.004) were positively correlated with microcalcification of thyroid nodules (Figure 8). Depression (OR = 3.860, 95%CI 1.052–14.161, p = 0.042) and FT4 (OR = 1.396, 95%CI 1.092–1.784, *p* = 0.008) levels were positively correlated with thyroid nodule aspect ratio > 1 (Figure 9). Depression (OR = 4.254, 95%CI 1.359–13.312, *p* = 0.013), TGAB positive rate (OR = 2.937, 95%CI 1.086–7.947, *p* = 0.034), TPOAB positive rate (OR = 2.937, 95%CI 1.086–7.947, *p* = 0.034) and FT4 (OR = 1.437, 95%CI 1.126–1.835, *p* = 0.004) level were positively correlated with the unclear boundary of thyroid nodules (Figure 10). Depression (OR = 4.134, 95%CI 1.810–9.439, *p* = 0.001), Stress (OR = 2.478, 95%CI 1.077–5.705, *p* = 0.033), Age (OR = 1.039, 95%CI 1.001–1.078, *p* = 0.041), and FT4 (OR = 1.242, 95%CI 1.033–1.493, *p* = 0.021) level were positively correlated with irregular edge of thyroid nodules (Figure 11).

## Discussion

The psychological and emotional issues in numerous populations during the COVID-19 pandemic have received increasing attention (Shi et al., 2020). During the COVID-19 pandemic period, the psychological status of the population was often a chronic stress status. A large number of people in China and even around the world experienced unprecedented negative emotions of anxiety and depression (Doglietto et al., 2020; Lai et al., 2020; Shi et al., 2020; Wang et al., 2020, 2021; Zhou et al., 2020; Chen et al., 2021; Harries et al., 2021; Hummel et al., 2021; Kassianos et al., 2021; Li et al., 2021; Mattila et al., 2021; Kim et al., 2022). Excessive psychological load and poor cognitive style could cause anxiety and depression, and then led to changes in the nervous endocrine and immune systems. Multiple endocrine organs, such as the pituitary, pancreas, adrenal, gonads, and thyroid gland, have also been affected by these negative emotions (Kazakou et al., 2021). Detrimental effects on thyroid function have been reported in patients with and without pre-existing thyroid disease. Most recently, thyroid autoimmune diseases have also been reported following Covid-19 vaccination (Jafarzadeh et al., 2022). COVID-19 vaccination was associated with a modest increase in antithyroid antibody titers. However, there was no clinically significant thyroid dysfunction post-vaccination (Lui et al., 2022). The benefits of COVID-19 vaccinations in terms of terminating the pandemic and/or reducing mortality rates can exceed any risk of infrequent complications such as a transient thyroid malfunction (Jafarzadeh et al., 2022). Emotional stress during the COVID-19 period affects the endocrine system. Levels of anxiety and depression were increased among pregnant women during this infectious pandemic. Thyroid function is altered during stressful experiences, and any abnormality during early pregnancy may significantly affect fetal development and pregnancy outcomes. Pregnant women in their first trimester in Shanghai during the COVID-19 outbreak were at an increased risk of having higher FT3 concentrations, lower FT4 concentrations, and isolated hypothyroxinemia (Lin et al., 2020). People in the northern area of Tianjin during the COVID-19 outbreak were at an increased risk of higher FT4, lower FT3, and lower TSH (Weiwei et al., 2022). A large number of studies had shown that negative psychological status was closely related to the occurrence and development of thyroid nodules, but the association between psychological status and ultrasonic manifestations of thyroid nodules had not been analyzed (Mizokami et al., 2004; Jonsdottir and Sjörs, 2019).

To the best of our knowledge, this was the first study to investigate the relationship between psychological health status and the ultrasonic manifestations of thyroid nodules at the peak of the COVID-19 epidemic. In this study, a cross-sectional study was conducted on the physical examination population in southwest China during the COVID-19 period, including 490 subjects, 243 of whom had thyroid nodules. The prevalence rate of 49.6% was higher than that in other regions (US Preventive Services Task Force et al., 2017), which may be related to dietary habits, living environment, iodine nutrition status, genetics, and other factors in southwest China.

This study concluded that old age, increased BMI, depression, anxiety, and stress were the independent risk factors for TNs. The average age of patients with TNs was higher than that without TNs, mainly over 40 years old, indicating that the prevalence of TNs increased significantly with age, which was consistent with most previous research results (Liang et al., 2020). The mechanism may be that with increasing age, the thyroid would undergo degenerative changes, which could lead to diffuse compensatory hyperplasia of the thyroid, resulting in thyroid nodules (Kwong et al., 2015). Some studies had shown that in Chinese individuals with more than adequate iodine intake, higher waist circumference was more strongly associated with higher BMI with an elevated risk of thyroid nodules. This might mean that adipose tissue in the waist area may influence the risk of thyroid nodules differently from adipose tissue elsewhere in the body. Central obesity lies at the core of metabolic syndrome (Alberti et al., 2005), so central obesity might be more closely associated with thyroid nodules than overweight and general obesity. A previous study showed that obesity is closely associated with thyroid cancer (Ma et al., 2015). Moreover, a meta-analysis study also showed that high BMI would increase the risk of thyroid cancer (Yin et al., 2018), and we found the same result in our population when we treated BMI as a categorical variable.

Dietary diversity was a proxy for micro-nutrient adequacy in diets (Ma et al., 2015), the association between iodine intake and thyroid disease presents a U-shaped curve, and iodine deficiency or excess would cause adverse effects, such as iodine deficiency could lead to hypothyroidism or increased prevalence of goiter, and excessive iodine salt intake increased the risk of thyroid nodules and thyroid cancer (Wang et al., 2021). On the contrary, Knudsen et al. found that the prevalence of goiter and single thyroid nodules in moderate and heavy drinkers was significantly lower than that in mild drinkers, which requires further proof. However, in our study, there was no significant correlation between drinking, iodine intake, and the occurrence of thyroid nodules. This result could not negate previous views, and further proof was needed by expanding the sample.

According to our findings, depression, anxiety, and stress were independent risk factors for TNs. Some scholars regard negative emotions as an independent risk factor for the occurrence of thyroid malignant tumors (Chen Fang et al., 2017). They believe that stress or negative emotions could cause changes in the cerebral cortex and hypothalamus, directly or indirectly weaken the immune system, and lead to an increase in the incidence of malignant nodules (Azizi and Malchoff, 2011).

In this study, elevated TSH levels, depression, stress, and chronic diseases (for example, hypertension, diabetes, etc.) were independent risk factors for thyroid parenchymal echo heterogeneity. Inhomogeneous thyroid echo was one of the manifestations of Hashimoto’s thyroiditis (an autoimmune disease), and its pathogenesis was associated with genetic and environmental factors. Hashimoto’s thyroiditis destroyed thyroid follicular cells, reduced thyroid hormone synthesis, and increased TSH due to negative feedback regulation. Four studies had reported that thyroid autoimmunity is related to depression and other negative emotions: a community sample (Carta et al., 2004), a primary health care sample (Bunevicius et al., 2007), a large number of pregnant women (Pop et al., 2006), and a large number of perimenopausal women (Pop et al., 1998).

There was a correlation between the ultrasonic characteristics of thyroid nodules and the benign and malignant thyroid nodules. In this study, the univariate analysis results with statistical significance in the ultrasonic characteristics of thyroid nodules were included in the multifactor logistic regression analysis to explore the risk factors. In the comparison between thyroid hypoechoic nodules and non-hypoechoic nodules, BMI, FT4, Tbil, Depression, Anxiety, and Stress were positively correlated with hypoechoic nodules. At present, there were few studies on the relationship between ultrasonic characteristics of thyroid nodules and psychological factors, and this study concluded that depression was positively correlated with microcalcification of thyroid nodules, nodule aspect ratio > 1, unclear nodule boundary, and irregular nodule edge. Stress was positively correlated with microcalcification and the irregular edge of thyroid nodules. Anxiety is positively correlated with the microcalcification of thyroid nodules. Overall, negative emotions were positively related to the ultrasonic characteristics of thyroid malignant nodules. Therefore, in this study, we speculated that negative emotions such as depression, anxiety, and stress may be the direct causes of determining the growth of nodules and the nature of nodules as benign or malignant.

Our research also showed that elevated FT4 levels were positively correlated with multiple thyroid nodules, hypoechoic nodules, nodule aspect ratio > 1, unclear nodule boundary, and irregular nodule edge. This may be closely related to the occurrence of thyroiditis. Previous studies had demonstrated that nodules with unclear boundaries and irregular border shapes had a higher risk of malignancy. It is well known that positive TPOAB and TGAB indicate the occurrence of the autoimmune disease Hashimoto’s thyroiditis. In this study, the positive rates of TPOAB and TGAB were also positively correlated with unclear boundaries of thyroid nodules.

Nevertheless, the results of the present work should be interpreted with caution in light of several limitations. The limitations of our study are that the initial design is a cross-sectional study, which could only indicate that depression, anxiety, stress, and other negative emotions may be the pathogenic factors of thyroid nodules and different abnormal ultrasound manifestations. Secondly, the study did not conduct cohort follow-ups of patients with thyroid nodules, so there was no direct evidence of the occurrence and development of thyroid nodules caused by negative emotions. Next, we will further increase the number of subjects to establish a cohort to further explore the impact of emotion on the ultrasound image characteristics of thyroid nodules.

## Conclusion

We found that there was a statistically significant association between anxiety, depression, stress, and the morbidity rate of thyroid nodules. While negative emotions also have a significant association with some abnormal ultrasound characteristics of thyroid nodules. Living in urban areas, negative emotions have been established as an important associated factor that was rarely used in previous research. The results of this study suggest that emotional screening and treatment for thyroid diseases should be conducted to potentially improve the prognosis of thyroid diseases. Especially in special periods such as COVID-19 infection, the negative emotions of the population may be more obvious. Enhanced training and integration of mental health treatment may reduce the occurrence of thyroid nodules. Further research with a larger sample size should be carried out to better assess the impact of anxiety and depression on the morbidity rate and characteristics of thyroid nodules.

## Data availability statement

The original contributions presented in the study are included in the article/supplementary material, further inquiries can be directed to the corresponding author.

## Ethics statement

The studies involving human participants were reviewed and approved by the ethics committee of The Second Affiliated Hospital of Chongqing Medical University (China). The patients/participants provided their written informed consent to participate in this study.

## Author contributions

ZL, ZH, and JD provided ideas for the design of the article, drafted the manuscript, and evaluated the quality of the study. ZL, ZH, YM, XQ, PY, YM, HC, GX, RB, and JD extracted the data and analyzed it. All authors contributed to the article and approved the submitted version.

## Funding

This work was supported by the Technological Innovation and Application Development Project of Chongqing, China (Grant No. cstc2020jscx-msxmX0060).

## Conflict of interest

The authors declare that the research was conducted in the absence of any commercial or financial relationships that could be construed as a potential conflict of interest.

## Publisher’s note

All claims expressed in this article are solely those of the authors and do not necessarily represent those of their affiliated organizations, or those of the publisher, the editors and the reviewers. Any product that may be evaluated in this article, or claim that may be made by its manufacturer, is not guaranteed or endorsed by the publisher.
